# Optimized Planar Microwave Antenna for Nitrogen Vacancy Center Based Sensing Applications

**DOI:** 10.3390/nano11082108

**Published:** 2021-08-19

**Authors:** Oliver Roman Opaluch, Nimba Oshnik, Richard Nelz, Elke Neu

**Affiliations:** Department of Physics, University of Kaiserslautern, Erwin-Schrödinger-Straße, 67663 Kaiserslautern, Germany; opaluch@rhrk.uni-kl.de (O.R.O.); nimba@rhrk.uni-kl.de (N.O.); richardnelz1203@hotmail.de (R.N.)

**Keywords:** diamond, nitrogen vacancy centers, microwave antenna, spin manipulation

## Abstract

Individual nitrogen vacancy (NV) color centers in diamond are versatile, spin-based quantum sensors. Coherently controlling the spin of NV centers using microwaves in a typical frequency range between 2.5 and 3.5 GHz is necessary for sensing applications. In this work, we present a stripline-based, planar, Ω-shaped microwave antenna that enables one to reliably manipulate NV spins. We found an optimal antenna design using finite integral simulations. We fabricated our antennas on low-cost, transparent glass substrate. We created highly uniform microwave fields in areas of roughly 400 × 400 μm^2^ while realizing high Rabi frequencies of up to 10 MHz in an ensemble of NV centers.

## 1. Introduction

Nitrogen vacancy (NV) centers in diamond each consist of a substitutional nitrogen atom and an adjacent lattice vacancy. In recent years, this crystal defect in its negatively charged form, NV${}^{-}$, has been extensively studied as a versatile spin-based quantum sensor (for simplicity, we use the term NV center for the negatively charged state throughout this manuscript). The NV center forms an electronic S = 1 spin system with a zero field splitting (ZFS) of 2.87 GHz between the $m_{s}$ = 0 and the degenerate $m_{s}$ = ±1 ground state. Manipulating these highly-coherent spin states is accomplished using microwaves (MW), e.g., via coherent oscillations (Rabi oscillations). These form the basis of spin-based quantum sensing using NV centers. An external magnetic field parallel to the NV’s high symmetry axis lifts the degeneracy between $m_{s}$ = ±1 states via the Zeeman effect, whereas a perpendicular field induces spin mixing. In addition, mechanical pressure, temperature and electrical fields interact with the NV spin, consequently making it a versatile atomic scale sensor [1,2,3,4,5,6,7,8]. To fully harness the NV’s sensing capabilities, providing spatially homogeneous, intense MW fields which are stable over long periods of measurement within well-defined volumes remains an on-going challenge. In this work, we investigate planar, micro-fabricated MW antennas to reliably deliver MW radiation to NV sensors.

NV centers are among the most promising candidates for many quantum technological applications due to their feasible spin initialization, read-out and manipulation using laser light (mostly around 532 nm) and MW fields [9]. An intersystem crossing in the NV center’s energy level scheme allows for spin state initialization by optical pumping and optical readout via spin dependent fluorescence [10,11]. The effect is known as optically detected magnetic resonance (ODMR, for details refer to [12,13]). ODMR using continuous MW is the most basic sensing mode using NVs (DC magnetometry). AC magnetometry employs pulsed MW sequences, e.g., spin-echo [14], or more advanced dynamical decoupling protocols [15,16,17,18,19,20].

Sensing with NVs can be typically divided into two approaches, either exploiting the atomic scale size of a single NV center to enable high resolution magnetometry, or sacrificing the latter in favor of highly sensitive magnetometry with an enhanced signal to noise ratio and enhanced sensitivity using an ensemble of NV centers. Sensing using ensembles requires highly homogeneous MW fields, as any field inhomogeneity over the ensemble constitutes a dephasing and consequently reduces sensitivity [21,22,23,24,25,26,27,28,29,30,31,32,33]. Additionally, for single NV-based schemes, spatially homogeneous fields are relevant, especially when scanning NV centers are used to image samples [13,34,35,36,37,38,39,40,41].

For AC magnetometry, the sensitivity depends on the coherence time $T_{2}$, which can be enhanced by employing dynamical decoupling protocols. In such multipulse protocols, short, intense MW pulses are of interest to perform full pulse sequences within the potentially low coherence and dephasing times of NV centers. This demand for homogeneous, stable and intense MW fields motivated our work for an optimized MW antenna design for NV sensing.

**Figure 1:**
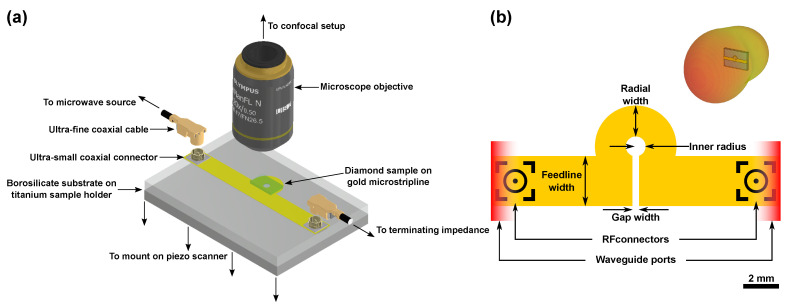
(**a**) A schematic representation of the Ω-shaped antenna and its implementation in a confocal microscope utilizing a piezo scanner (coaxial connector design by avitek, coaxial cable design by Steven Minichiello and objective design by thorfynn; source: https://grabcad.com accessed on 7 June 2021). (**b**) A schematic representation of the geometry of the Ω-shaped antenna design (note that the scale bar is approximate and the sketch does not give the exact geometry of the simulated antenna but illustrates the design in general). Microwave (MW) modes enter and leave the calculation domain via the waveguide ports indicated in red. Inset: Simulated MW radiation pattern. Here, strong directivity towards the sample is visible.

In this study, we investigate MW antenna designs compatible with confocal and atomic force microscopy (AFM) based sensing applications with NV centers (Figure 1a) [13,34,35,36,37,38,39,40,41]. Commonly used antenna concepts have design-related limitations with respect to these applications: Wire antennas suffer from inhomogeneity in the radiated fields requiring precise positioning [42]. Stripline antennas tend to be large in size and overload piezo scanners in confocal microscopes when placed on top [43,44,45,46]. Coil antennas only radiate MW of low amplitude [47,48,49]. Resonators are limited in bandwidth [50,51,52,53,54,55,56].

We aimed for planar antenna designs that can be efficiently incorporated into sensing setups without significant hardware modifications (Figure 1a). To investigate larger microstructures, e.g., biological samples with characteristic dimensions of the order of several hundred μm, one aims for macroscopically large and homogeneously radiated MW fields of high amplitude. The stripline-based design we made offers risk-free sample handling by mounting the sample on top of the antenna, implementation without blocking optical access and the advantage of established fabrication technology. By using transparent substrates for the antenna, we provide an antenna design that also allows observation in inverted geometries where the optical path crosses the antenna or the antenna is attached to the microscope objective [25,57]. We made a design based on an Ω-shape (Figure 1b). This layout inherently leads to the radiation of a homogeneous MW field within the aperture towards the designated sample volume (see inset Figure 1b).

Our manuscript is structured as follows: Section 2 summarizes the process flow, relevant materials and parameters for our MW antenna fabrication. Section 3 summarizes the methodology, assumptions and numerical simulation approach to find an optimized antenna design. Section 4 briefly describes the experimental setup and methods to characterize and test the antennas. Section 5 discusses our experimental findings on the MW antenna performance.

## 2. Microfabrication Methods

**Figure 2:**
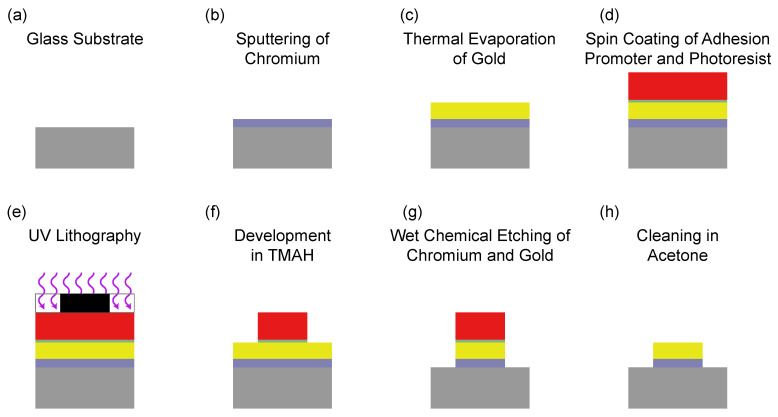
Process flow for Omega antennas. The color key: grey—glass substrate; blue—chromium; yellow—gold; green—adhesion promoter; red—photoresist; black—photomask; violet—UV-illumination.

We fabricated the antennas on borosilicate glass substrates (relative permittivity $\epsilon_{r}$ = 4.82, substrate thickness $s_{z}$ = 1 mm; Figure 2a). To prepare thin film deposition, we cleaned the substrate in acetone followed by isopropanol using an ultrasonic bath. We removed absorbed water by heating the substrates on a heatplate (120 ${}^{\circ }$C, 10 min). We then used either sputtering or thermal evaporation to deposit an adhesion layer (20 nm chromium) (Figure 2b) followed by a 100 nm gold layer (Figure 2c). To fabricate the antenna structure, we used spin coating (6000 rpm, 1 min) followed by baking (120 ${}^{\circ }$C, 2 min) to deposit an adhesion promoting sub-monolayer of TI Prime (MicroChemicals, Ulm, Germany). We then spin coated the photoresist AZ1518 (6000 rpm, 1 min, Merck, Darmstadt, Germany) (Figure 2d). After a prebake (100 ${}^{\circ }$C, 50 s), we applied contact UV lithography using a laser written binary intensity amplitude chromium photomask (Figure 2e). Lithography parameters were chosen according to resist manufacturer specifications resulting in an area dose of 33.6 $\frac{\mathrm{mJ}}{{\mathrm{cm}}^{2}}$. We developed the resist mask by stirring the exposed substrates in TMAH (Acros Organics, Fair Lawn, NJ, USA) 2.5% in water (Figure 2f). After rinsing it in monodistilled water, we performed a postbake (120 ${}^{\circ }$C, 50 s). To form the antenna structure using wet chemical etching: First, we stirred the substrate in gold etchant (Sigma-Aldrich, St. Louis, MI, USA) followed by rinsing it in chromium etchant (Sigma-Aldrich, Figure 2g). To remove residual photoresist and potential contamination, we stirred the fabricated MW antenna in acetone and isopropanol (Figure 2h). This allowed electrical contacting by gluing SMT ultra-miniature coaxial connectors (U.FL-R-SMT(01), Hirose Electronic, Tokyo, Japan) using electrically conductive epoxy adhesives (EPO-TEK H20E, Epoxy Technology, Billerica, MA, USA).

## 3. Numerical Simulation and Optimization

For the numerical optimization, we used the time domain solver of the commercial software CST Microwave Studio (Dassault Systèmes, Vélizy-Villacoublay, France), which uses the finite integral technique (FIT) to solve the Maxwell equations [58]. We defined waveguide ports at the ends of the microstripline, where waveguide modes could leave or enter the domain of interest. During each simulation, the total MW power delivered at the excited port was 500 mW. We typically ran the simulation in the frequency range between 2 and 4 GHz. To enhance far field calculation accuracy, our simulation included additional space between the structure and the simulated space boundaries ($\frac{\lambda_{\mathrm{MW}}}{4}\approx$ 26 mm). The boundaries fulfilled the perfectly matched layer (PML) condition, assuring that incident waves were being absorbed with negligible back reflections. The applied mesh exhibited variable mesh cell sizes and was locally refined by introducing additional cells within the space around critical structural features, such as corners, edges and material interfaces. This approach allows for highly precise simulations while keeping computational time reasonable. Due to the varying structure geometries, the simulated space was typically meshed with approximately 180 × 190 × 140 cells, resulting in 4.6 million cells involving a total volume of roughly 68 mm × 63 mm × 54 mm.

The linear and radial parts of the antenna form capacitive and inductive components, respectively. Altogether, they determine the antenna’s resonance. To harness this antenna design for NV-based sensing, we optimized the linear and radial geometry to match antenna resonance and NV spin transition frequency. Consequently, the main parameters to be optimized were the gap width $g_{w}$ and the radial width $r_{w}$ of the Ω-antenna (see Figure 1b). In addition, optimizing the feedline width $f_{w}$ also yielded a slight improvement. We also considered the thickness of the gold layer $t_{\mathrm{Au}}$ forming the conductor. Our results indicate a negligible influence on resonance frequency and field distribution for films thinner than 1 μm. Furthermore, we did not observe significant improvement capabilities by increasing the layer thickness further. All simulated gold layers are thinner than the theoretically expected skin depth for gold of 1.4 μm at 2.87 GHz, leading to an approximately homogeneous current density throughout the gold layer. We assume the contribution of the chromium adhesion layer to the antenna properties to be negligible. We thus did not vary the thickness of this layer and set it to 20 nm.

**Figure 3:**
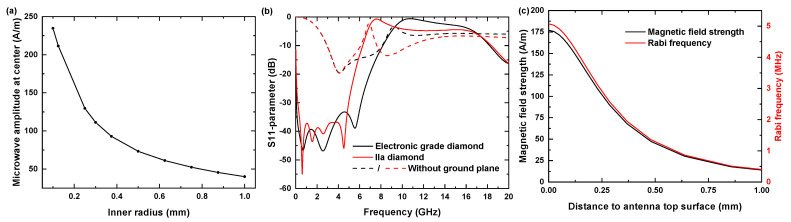
(**a**) Achievable MW amplitude for different inner radius values $r_{w}$. Points from different simulation runs. (**b**) Calculation of the frequency-dependent $S_{11}$ parameter (back reflection coefficient) with a ground plane for the 50 μm thick diamond (solid black) and the 300 μm thick diamond (solid red) and without the ground plane (dashed lines). (**c**) MW amplitude and Rabi frequency dependence on the distance to the antenna top surface. Rabi frequencies $\Omega_{R}$ were determined for a resonantly driven transition of the subset of nitrogen vacancy (NV) centers aligned in 〈111〉 crystallographic direction. Input MW power equals 1 W.

To achieve a high MW field within the aperture, the inner radius of the aperture should be minimized, contradicting the aim for a macroscopically large radiated area. For all optimization steps, we evaluated the magnetic field amplitude at the center of the antenna’s aperture at a distance of 10 nm below the diamond sample’s top surface, which corresponds to the approximate depth of shallow NV centers. For the diamond sample, we considered a (100)-oriented diamond (2.5 mm × 1.5 mm × 50 μm). Typically, for experiments using single NV centers, high purity electronic grade, chemical vapor deposited diamonds (substitutional nitrogen content <5 ppb, manufacturer: Element Six, Didcot, United Kingdom) were used. For testing the antenna, we used less pure diamonds (substitutional nitrogen content <1 ppm, termed IIa diamonds) which were 300 μm thick and contained dense ensembles of NV centers, which we also considered for better comparison with our experimental results. Note that for the simulations, the dielectric constant used for electronic grade and IIa diamonds was identical, as it was not influenced by the nitrogen content. By varying the inner radius of the aperture, we found a trade-off between the achievable MW amplitude and the radiated area size, as shown in Figure 3a. We chose the inner radius to be 300 μm, which was smaller than the diamond under consideration, but allows one to investigate reasonably large sample areas. Note that only for an inner radius value of 300 μm, the design was fully optimized. For all other values, we expected the amplitude could be further enhanced subjecting to the repeated optimization of the remaining parameters. Otherwise, these calculations assumed the same parameter values we determined during the optimization for an inner radius of 300 μm.

We set the lateral substrate size to 16 mm × 11 mm. This substrate allowed for straightforward mounting to typical piezo-scanner in confocal microscope setups. The simulation also included the sample holder which we assumed to be a 24 mm × 15 mm titanium plate, as compliant with the piezo scanner systems (attocube systems, Haar, Germany) used in our setup.

For the optimization, we applied the software’s internal goal function by defining quantities and frequency ranges in which they were evaluated, type of goal (minimization/maximization) and weight factors. We consider different aspects relevant for the antenna performance. The predominant limitation in maximizing the radiated field amplitude was given by a frequency mismatch leading to significant back reflections of the input MW signal characterized by the high frequency circuitry component’s S${}_{11}$-parameter. In accordance with other reported work, we minimized the S${}_{11}$-parameter of the antenna and consequently the back reflection of the input signal at 2.87 GHz [45,46,47,51,52,53,54,56]. Optimizing the Ω-antenna for NV sensing involving variable, high magnetic bias fields, and for obtaining complete ODMR spectra, required us to maximize the antenna bandwidth. To account for that, we extended the minimization of S${}_{11}$ to the frequency range between 2.77 and 2.97 GHz. Overall, we performed two evaluations of S${}_{11}$, once individually at 2.87 GHz, and again in the range between 2.77 and 2.97 GHz. Both the evaluations were given equal weight, allowing us to maximize the bandwidth and simultaneously favor a centering of the resonance at 2.87 GHz. Furthermore, our optimization goal function aimed to maximize the magnetic field strength. The weight factor was chosen accordingly to allow for equal contributions between the S${}_{11}$-parameter and the target microwave amplitude.

Under these assumptions, we determined the following set of optimized geometry parameters: gap width $g_{w}=7$ μm, radial width $r_{w}=1.151$ mm, feedline width $f_{w}=1.851$ mm. This antenna structure exhibits multiple resonances at 0.7, 2.6 and 5.5 GHz. For the most relevant resonance at 2.5 GHz, we found a value of −47 dB, as illustrated by the $S_{11}$-parameter curve in Figure 3b. For the optimized case of 50 μm thick electronic grade diamonds, the bandwidth for this structure amounts to 8.2 GHz, enabling reliable NV spin manipulation involving bias fields up to 190 mT. For 300 μm thick IIa diamonds, our results indicate a lowering of the bandwidth down to 6.3 GHz, consequently limiting feasible bias fields to 120 mT. Our titanium sample holder acted as a ground plane and significantly decreased back reflections. To estimate the Ω-antenna’s robustness against geometry deviations arising from UV lithography tolerance errors, we repeated the simulation and adjusted either the gap width by ±1 μm or the radial width by ±10 μm and compared the respective $S_{11}$-parameter curves. We found slight resonance shifts of approximately −50 MHz/μm for increasing gap width and −20 MHz/μm for increasing radial width. Together with its wideband resonance property, this design is suited for versatile applications in the field of NV-based sensing [21,23,25,26,27,28,29,30,31,32,33,34,35,36,38,39,40,41].

**Figure 4:**
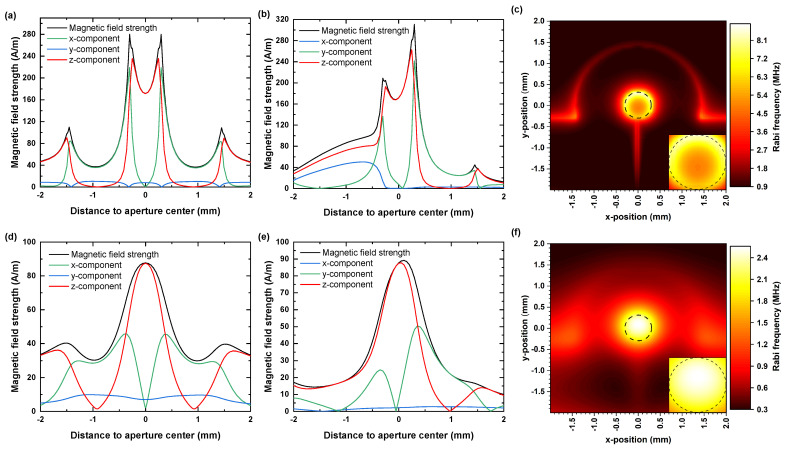
Field simulation results for a 50 μm thick electronic grade diamond (**a**–**c**) and for a 300 μm thick IIa diamond (**d**–**f**). All field data were taken 10 nm below the diamond sample’s top surface and correspond to a MW input power of 1 W. (**a**,**b**,**d**,**e**) Field distribution of the MW in x and y directions. (**c**,**f**) 2D maps of theoretically expected Rabi frequencies $\Omega_{R}$ for a resonantly driven transition of the subset of nitrogen vacancy (NV) centers aligned in 〈111〉 crystallographic direction. The dashed circle marks the circumference of the aperture. Insets: detailed views of the aperture areas.

With increasing distance to the antenna’s top surface we found a decrease in MW field amplitude (see Figure 3c). For NV-Centers very close to antenna’s top surface, the achievable Rabi frequencies at the aperture center reached values of up to 5.1 MHz. For coherent spin manipulation of shallow NV centers, we found in 50 μm electronic grade diamonds and 300 μm thick IIa diamonds Rabi frequencies of 4.9 and 2.5 MHz. Due to an approximate radial symmetry of the Ω-antenna, the established MW field distribution shows a radial distance dependence from the aperture center in the x-y plane (see Figure 4a,b). For an electronic grade diamond with 50 μm thickness, the typically achieved value of the magnetic field amplitude exceeded 170 $\frac{A}{m}$. Towards the aperture circumference, we observed a parabolic increase up to 280 $\frac{A}{m}$. Within an area with a radius of 260 μm, the total MW field is mainly given by its perpendicular z-component, as shown in Figure 4a,b. Beyond the circumference, there is a further increase in the amplitude due to a strong increase in the lateral field components, in contrast the z-component drops off quickly. In the direction of the gap, the MW field amplitudes do not reach the mentioned peak values and decrease near it instead. From the field distribution, we calculated the expected Rabi frequency $\Omega_{R}$ considering detuning due to field components parallel to the NV axis for one subset of NV centers (see Figure 4c). For a square area fitting into the aperture (edge length 420 μm), we determined an average value for the expected $\Omega_{R}$ of 5.9 MHz ± 0.5 MHz. For later comparison with our experimental data, Figure 4d–f shows the simulation data for a IIa diamond of 300 μm thickness. In that case we determined an average value for the expected $\Omega_{R}$ of 2.38 ± 0.06 MHz.

## 4. Experimental Setup and Methods

To asses the performances of the microfabricated antennas, we applied them to typical methods used in NV-based sensing (see [12,13]). We mounted the antenna on a sample holder using a thin layer of Crystal Bond 509 (Structure Probe Inc, West Chester, PN, USA). As a sample, we used a single crystalline, (100)-oriented IIa, chemical vapor deposited diamond (Element Six, 3 mm × 3 mm × 300 μm, substitutional nitrogen content <1 ppm). We cleaned the diamond in a tri-acid mixture (H${}_{2}$SO${}_{4}$ 96%, HClO${}_{4}$ 70%, HNO${}_{3}$ 65%) at 500 ${}^{\circ }$C for 1 h. The sample contained a homogeneous ensemble of native NV centers, as confirmed by multiple photoluminescence confocal scans.

We used a laboratory-built confocal microscope setup (numerical aperture NA = 0.8, excitation wavelength $\lambda_{Laser}$ = 532 nm). Confocal filtering of the NV ensemble’s fluorescence was realized by using a single mode optical fiber. To measure the fluorescence, we used a single photon detector (SPCM-AQRH-14, quantum efficiency ≈ 68%, Excelitas Technologies, Waltham, MA, USA) and a data acquisition card for acquiring and logging the signal (PCIe-6323, National Instruments, Austin, TX, USA).

We connected the Ω-antennas to an MW source (SG 384, Stanford Research Systems, Sunnyvale, CA, USA) with an amplifier (ZASWA-2-50DR+, typ. +30 dB, Mini Circuits, Brooklyn, NY, USA). We applied a static bias field by positioning a neodymium (NdFeB) permanent magnet using a three-axis linear stage. We aligned the bias field with one of the 〈111〉 crystal directions within an error of $\approx 15{\;}^{\circ }$.

We realized Rabi oscillations (see [59]), where we determined the achievable Rabi frequency $\Omega_{R}$ in the NV spin system with the MW power deployed by the antenna. The $\Omega_{R}$ is the prime measure for antenna performance because it depends linearly on the perpendicular component of the MW’s amplitude relative to the NV axis. These measurements were done for different positions to map the spatial homogeneity of the Rabi frequency. We also acquired continuous wave and pulsed optically detected magnetic resonance (ODMR) measurements (see [60]) and ran dynamical decoupling protocols (see [61]) showing the suitability of the MW antenna design for nanoscale NV-based sensing applications.

To generate the control MW pulses used in dynamical decoupling experiments, we used the in-built digital I/Q mixer of the MW signal generator in combination with external quadrature signal sources (Pulse Streamer 8/2, Swabian Instruments, Stuttgart, Germany). With this approach, we controlled the pulse duration, shape and polarity of the MW signal. For dynamical decoupling experiments, we used a stronger amplifier (ZHL-16W-43-S+, typ. +45 dB, Mini-Circuits) and a 520 nm diode laser (DL nSec, PE 520, Swabian Instruments ).

## 5. Antenna Performance

Comparison—Sputtering and Thermal Evaporation: We investigated the influence of the physical vapor deposition (PVD) method used to deposit the electrically conducting gold layer. For this purpose, we characterized two geometrically identical MW antenna batches, one with a sputtered gold layer the other with a thermally evaporated gold layer. We note that the sputtered gold layer showed superior adhesion compared to the thermally evaporated one.

**Figure 5:**
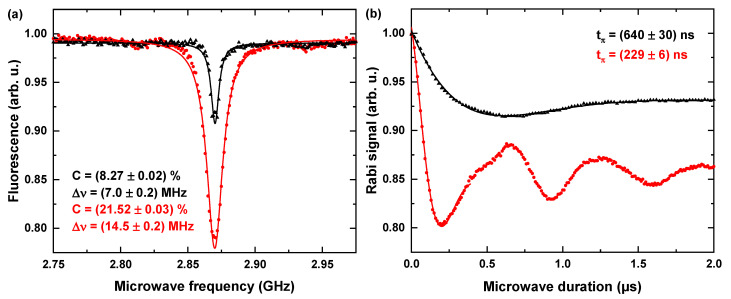
Comparison between sputtered and evaporated layers. The evaporated antennas show better performance in terms of radiated MW amplitude, as shown by the increased optically detected magnetic resonance (ODMR) contrast C ((**a**), 8% vs. 22%), and Rabi frequency $\Omega_{R}$ ((**b**), 800 kHz vs. 2.2 MHz, $t_{\pi }$ = π-pulse duration). Note that the ODMR resonance of the evaporated antenna is strongly power broadened (Δν = FWHM). Points indicate the experimentally obtained data and solid curve denotes fits used to obtain the mentioned parameters.

We performed ODMR and Rabi oscillations without an external magnetic field. The optical excitation power was set to $P_{\mathrm{Opt}}$ = 700 μW and the MW power before amplification (+45 dB) was $P_{\mathrm{MW}}$ = −15 dBm. We found that antennas with a thermally evaporated gold layer outperformed antennas with a sputtered layer, as shown in Figure 5a. On average, we determined ODMR contrasts of 10% ± 3% and $\Omega_{R}$ of 0.86 ± 0.06 MHz for sputtered antennas; and 21% ± 2% contrast and $\Omega_{R}$ of 2.4 MHz ± 0.5 MHz for thermally evaporated ones. The thermally evaporated antennas achieved comparably high contrasts in comparison to highly optimized ODMR experiments (≈30% according to [62,63]). Significant power broadening of the ODMR resonances indicates high MW power driving the NV centers, as displayed in Figure 5b. We attribute this enhancement to a lower power loss within the gold layer due to decreased amounts of impurities and crystal defects created during the sputtering process. Taking into account this finding, we restricted further characterization to thermally evaporated antennas.

**Figure 6:**
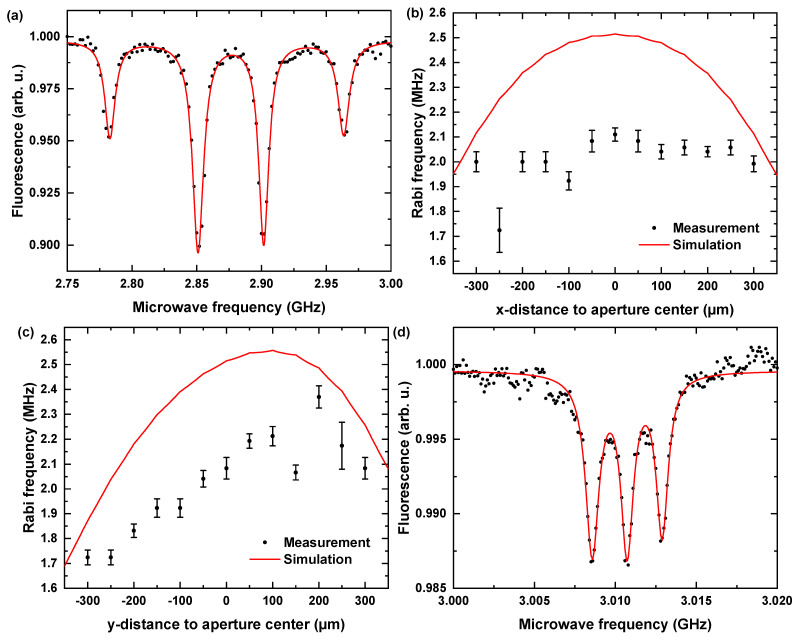
(**a**) Continuous ODMR measurements of an NV ensemble with an externally applied magnetic field. For the outermost resonance pair, a splitting of 181 MHz ± 1 MHz with high contrast (4.67% ± 0.06% at −15 dBm source MW power) was obtained. For the corresponding subset of NV centers, we determined a projection of the magnetic field on the NV axis of 6.45 mT ± 0.05 mT. (**b**,**c**) Characterization of the homogeneity of the radiated MW field amplitude perpendicular and parallel to the gap. While the amplitude parallel to the gap slightly depended on the distance to the gap, as expected, the amplitude perpendicular to the gap remained unaffected, showing that the full area of the aperture is reliably usable for spin manipulation protocols. (**d**) Pulsed ODMR of one of the resonances showing the hyperfine transitions due to ${}^{14}N$ nuclear spin coupling. The black points indicate the experimentally obtained data, solid black lines indicate the error bars and the solid red lines indicate either fits or simulated data.

Spatial Homogeneity: To evaluate the spatial homogeneity of the radiated MW field, we performed Rabi oscillation measurements ($P_{\mathrm{Opt}}$ = 700 μW, $P_{\mathrm{MW}}$ = −15 dBm) along multiple designated line-cuts in x and y directions (perpendicular/parallel to the gap). For these measurements, we applied an external magnetic field mostly aligned with one of the 〈111〉 crystallographic directions, leading to a significant Zeeman splitting for that subset of NV centers [13]. In this situation, ODMR contrast in a non-aligned ensemble intrinsically lowers. For this resonance pair, we observed 4.67% ± 0.06% contrast, which is typical for NV ensembles involving not perfectly aligned bias fields, leading to spin-mixing and charge state instabilities [62,63]. Rabi oscillations were driven typically at a transition frequency of 2.783 GHz, as in Figure 6a corresponding to a bias field strength of $B_{\mathrm{NV}}$ = 6.45 mT ± 0.05 mT. Figure 6b,c summarizes the measurements of the spatial homogeneity of the $\Omega_{R}$. Line-cuts perpendicular to the gap reveal constant $\Omega_{R}$ within experimental errors. As expected from the simulation results, we observed a slight increase in $\Omega_{R}$ with increasing distance from the gap opening. We determined an average value of 2.1 ± 0.1 MHz over all measurement points within 200 μm distance to the aperture center. Homogeneity and range are in good agreement with our simulation results (compare Figure 4f). We attribute the on average slightly lowered Rabi frequency $\Omega_{R}$ to unconsidered losses of our MW circuitry and the potential impedance mismatch of the electrically conducting gluing point for the coaxial connectors. The radiated MW fields’ homogeneity on the scale of the aperture area enables higher coherence times for NV ensembles, leading to enhanced sensitivities. Furthermore, simultaneous MW irradiation of high amplitude within a macroscopic area provides position independent high contrast and therefore an improved signal-to-noise ratio, making this antenna type promising for wide-field [21,22,23,24,25,26,27,28,29,30,31,32,33] and scanning NV-based sensing applications [13,34,35,36,37,38,39,40,41].

Suppression of power broadening-pulsed ODMR: NV-related applications often require one to suppress the power broadening—e.g., when hyperfine transitions of the NV center need to be addressed. Pulsed ODMR schemes instead of continuous driving can suppress power broadening [64]. Here, we show that our antenna also allows for reliable pulsed ODMR measurements. We performed pulsed ODMR using NV centers close to the aperture center. Optical spin initialization and read out at 532 nm were realized with 1 μs long laser pulses. Resonant spin manipulation involved 2 μs MW pulses at −30 dBm source MW power amplified by factor of +45 dB. The pulsed ODMR measurement at $B_{\mathrm{NV}}$ = 5 mT in Figure 6d reveals the hyperfine interaction of the NV center electronic spin with the ${}^{14}$N nucleus. We thus confirm the usability of our antennas at low power for efficient, pulsed spin manipulation.

**Figure 7:**
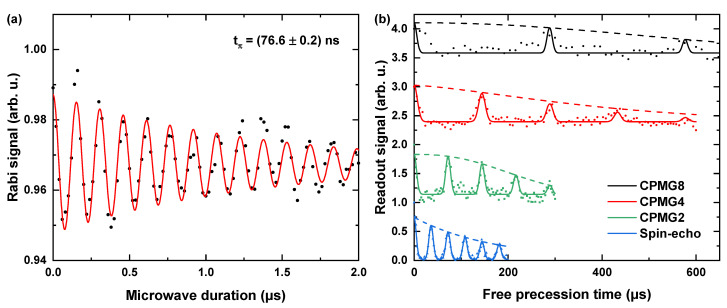
(**a**) Typical Rabi oscillation measurements, which are primarily used to obtain relevant pulse parameter to be used in multi-pulse sensing schemes. The measurement performed at $\Omega_{R}\approx$ 6.5 MHz. (**b**) Spin-echo and Carr Purcell Meiboom Gill (CPMG)-n measurements with the NV ensemble performed with the pulse parameters obtained from (**a**). The plots have been fit and scaled along the y-axis for comparison. The points show the experimentally obtained data, solid curves denote the fit and the dashed lines indicate the fit envelopes. The spin-echo measurements reveal $T_{2}\approx$ 167 μs. Applying CPMG-8 pulse sequences leads to almost a fourfold improvement in the decoherence time of the spin state. From the fit, the calculated enhanced decoherence times for the CPMG-2/4/8 protocols were ≈245 μs/≈425 μs/≈638 μs, respectively.

High-Power Applications: High MW powers enable us to realize faster and more complex pulse sequences typically occurring in dynamical decoupling schemes [20]. Dynamically decoupling the NV center from the environmental noise can increase the coherence times, resulting in higher AC magnetic field sensitivities and an increase of the detectable frequency range of AC magnetic fields. Here, we demonstrated dynamical decoupling using spin-echo [14] and Carr Purcell Meiboom Gill (CPMG) pulse sequences [15]. We resonantly drove a transition at 3.01076 GHz corresponding to $B_{\mathrm{NV}}$ = 5 mT. Note that we did not observe any significant heating or evident nonlinear effects when applying high-power MW pulses. We observed $\Omega_{R}$ in the range of 6 to 10 MHz, depending on the control power amplification (Figure 7a). With the MW power applied in Figure 7a, we realized spin-echo and CPMG pulse sequences with different numbers of π-pulses (1, 2, 4 and 8) (Figure 7b). In comparison to spin-echo, which yielded $T_{2}$ = 167.1 μs, CPMG8 allowed us to increase $T_{2}$ up to 638.1 μs. The antenna is evidently suitable for high-power applications, especially for NV-based AC magnetometry. We infer that our antennas are also usable for advanced spin manipulation techniques based on quantum optimal control theory [65].

## 6. Conclusions

In this work, we designed and fabricated MW antenna systems for NV-based sensing applications. We optimized a Ω-shaped, microstripline design considering macroscopic spatial homogeneity, wide bandwidth, large amplitudes and ease of implementation with scanning confocal/AFM setups, risk-free sample handling and straightforward fabrication. Our simulations showed that the gap and the radial structure of the antenna govern its resonance properties. A key feature of the design is an aperture with a radius of 300 μm, defining the homogeneously irradiated area. The design achieves a high bandwidth, along with desirable resonance properties within a macroscopic area with considerably high MW field amplitudes well-suited for NV-based sensing applications. We established a reliable microfabrication process and tested the fabricated MW antennas for typical NV spin manipulation protocols.

We compared sputtering and thermal evaporation for the deposition of the electrically conducting gold layer and found that thermally evaporated layers significantly improve the antenna performance. By mapping the achievable Rabi frequencies within the antenna aperture, we showed the macroscopic homogeneity of the radiated MW field. We tested these antennas in low-power and high-power applications by demonstrating pulsed ODMR measurements and advanced dynamical decoupling protocols. Our antennas can be straightforwardly used in optimized NV sensing approaches, e.g., using optimal control pulses, and in scanning NV magnetometry setups. Consequently, we envisage that our antenna design can add to practical NV sensing.

## Data Availability

The data presented in this study are available on request from the corresponding author.
